# Adaptive policies balancing trade, productivity and cropland increases can support Zambia’s nutrition security under future climate shocks

**DOI:** 10.1038/s43016-026-01405-1

**Published:** 2026-07-28

**Authors:** Stewart Jennings, Andrew Challinor, Jennie I. Macdiarmid, Richard King, Edward Pope, Stephen Whitfield, Rebecca Sarku, Christian Chomba, Masiye Nawiko, Shiluva Chauke Nkanyani, Graham Horgan, Jon Hellin, Mary Ng’endo, Eleanor Fisher, Liangzhi You, Anne Timu, Grazia Pacillo, Giulia Caroli, Anna Belli, Pedro Anglaze Chilambe, Evan Girvetz, Sabrina Rose, Giriraj Amarnath, Alan Kennedy-Asser, Richard Rigby, Ana Maria Loboguerrero

**Affiliations:** 1 Institute for Climate and Atmospheric Science, School of Earth, Environment and Sustainability, University of Leeds, Leeds, UK; 2 The Rowett Institute, School of Medicine, Medical Sciences and Nutrition, University of Aberdeen, Aberdeen, UK; 3 Chatham House, The Royal Institute of International Affairs, London, UK; 4 Hadley Centre, Met Office, Exeter, UK; 5 Sustainability Research Institute, School of Earth, Environment and Sustainability, University of Leeds, Leeds, UK; 6 Agricultural Consultative Forum, Lusaka, Zambia; 7 The Food Agriculture and Natural Resources Policy Analysis Network, Pretoria, South Africa; 8 Biomathematics and Statistics Scotland, Aberdeen, UK; 9 International Rice Research Institute, Manila, Philippines; 10 Leibniz Centre for Agricultural Landscape Research, Müncheberg, Germany; 11 International Rice Research Institute, Nairobi, Kenya; 12 The Nordic Africa Institute, Uppsala, Sweden; 13 International Food Policy Research Institute, Washington, DC USA; 14 International Center for Tropical Agriculture, Cairo, Egypt; 15 International Center for Tropical Agriculture, Pretoria, South Africa; 16 Food and Agriculture Organization of the United Nations, Rome, Italy; 17 International Center for Tropical Agriculture, Nairobi, Kenya; 18 Bioversity International, Rome, Italy; 19 International Water Management Institute, Colombo, Sri Lanka; 20 School of Geographical Sciences, University of Bristol, Bristol, UK; 21 Cabot Institute for Environment, University of Bristol, Bristol, UK; 22 Centre for Environmental Modelling and Computation, School of Earth, Environment and Sustainability, University of Leeds, Leeds, UK

**Keywords:** Agriculture, Climate-change impacts, Climate-change adaptation, Risk factors

## Abstract

Policies in sub-Saharan Africa are constrained by a limited knowledge of climate change extremes and a focus on agricultural production rather than nutrition supply. Here we model the impacts of future extremes on national-level calorie and nutrient supply in Zambia for production and nutrition focused policy scenarios. We identify the specific cropland, yield and import increases required to achieve climate-resilient nutrition security and highlight policy options.

## Abstract (web summary)

Sub-Saharan Africa’s food systems are increasingly vulnerable to climate change. Using the integrated Future Estimator for Emissions and Diets (iFEED) framework, Zambian nutrition security and policy options are explored under average and extreme climate scenarios.

## Main

Inadequate food supplies triggered by climate change may put millions at risk of nutrition insecurity in sub-Saharan Africa (SSA) by 2050^1^. SSA is highly vulnerable to increasing climate extremes due to high present-day temperatures, heavy reliance on rainfed agriculture and relatively weak capacity for adaptation. The challenges facing Zambia are typical of SSA: intensifying climate extremes, with rainfall and temperature extremes projected to increase in Zambia by 2050^2^; a rapidly growing population; stagnating crop productivity (despite gradual improvements in Southern Africa^3^, average Zambian maize yields in the 2020s are similar to those of the previous decade)^4^; an overreliance on maize; and underdeveloped agricultural infrastructure. Zambia currently faces nutrition insecurity, with an inadequate supply of food resulting in multiple micronutrient deficiencies including iron, calcium, zinc, folate, fibre, riboflavin and vitamin A^2,5^.

Adaptation gaps exist when adaptation implementation fails to meet adaptation needs, and are composed of gaps in knowledge and capacity, including finance^6,7^. Typically, long-term climate change impact studies assess how future crop yield or production will change with average changes in climate^8^, but knowledge gaps remain. Sometimes the supply of calories is considered^9^, but macro- and micronutrient requirements are rarely taken into account^10^. While some studies point to nutrient shortfalls by mid-century^10,11^, they omit extreme climate impacts; conversely, studies accounting for climate extremes focus on production or calories rather than nutrient supplies^12,13^. Thus, results so far, for example those indicating that over one-third of the population could be at risk of hunger by mid-century^14^, lack any kind of assessment of the impact of extreme climate years on population-level macro- and micronutrients and the adaptation strategies that could deal with these impacts.

Policy approaches to climate and food systems reflect previous academic studies in that they focus on incremental adaptive coping mechanisms, relatively few agricultural commodities, productivity growth and mean shifts in climate^15,16^. This contrasts with the larger structural changes necessary for improved nutrition in the face of climate extremes. This study, therefore, assesses contrasting transformative changes to agriculture and the adequacy of these changes for calorie and nutrient supplies to meet population-level dietary requirements (hereafter referred to as nutrition security) considering extreme climate impacts. In doing so, we help to bridge critical knowledge gaps in both research and policy.

We use an integrated assessment framework—the integrated Future Estimator for Emissions and Diets (iFEED^17^)—to assess Zambian nutrition security in extreme future years. We explore two contrasting future scenarios. The ‘nutrition focus’ scenario diversifies domestic crop production using the smallest land expansion possible, targeting population-level nutrient requirements in typical future years, and explores impacts on nutrition security in extreme future years. This scenario has no expansion of livestock pasture and minimal cropland expansion, promoting nutrient-adequate diets while reducing the negative environmental impacts associated with expansion^18^. The contrasting ‘production focus’ scenario seeks to maximize food production on all available land without altering crop diversification and is agnostic to nutrition security outcomes, thus reflecting a business-as-usual policy approach.

We seek to explore the adequacy of the two scenarios for ensuring resilience to the largest plausible future climate extremes. We therefore drive the iFEED framework with a subset of bias-corrected climate data for Africa^19^, representing the largest shocks to crop production by mid-century (2040–2060) under Representative Concentration Pathway 8.5 (RCP8.5; see Methods for more details). We focus on RCP8.5, and this subset, to explore how food systems might adapt to the plausible worst-case production shocks in this crop–climate ensemble. For each scenario, we assess nutrient supplies in an average future year and an extreme future year that represents the simultaneous largest possible production shocks of each crop. We assume that future international food imports and exports remain in the same proportions to domestic production as in the historical baseline. Both scenarios also assume no changes to irrigated areas. After calculating crop production, livestock production is calculated as a fixed function of livestock feed and assumes that livestock productivity remains constant in the future. We explore options for achieving nutrition security given these food production changes with these extreme climate impacts and finally make policy recommendations that bridge the gaps between current policies and future requirements.

**Fig. 1:**
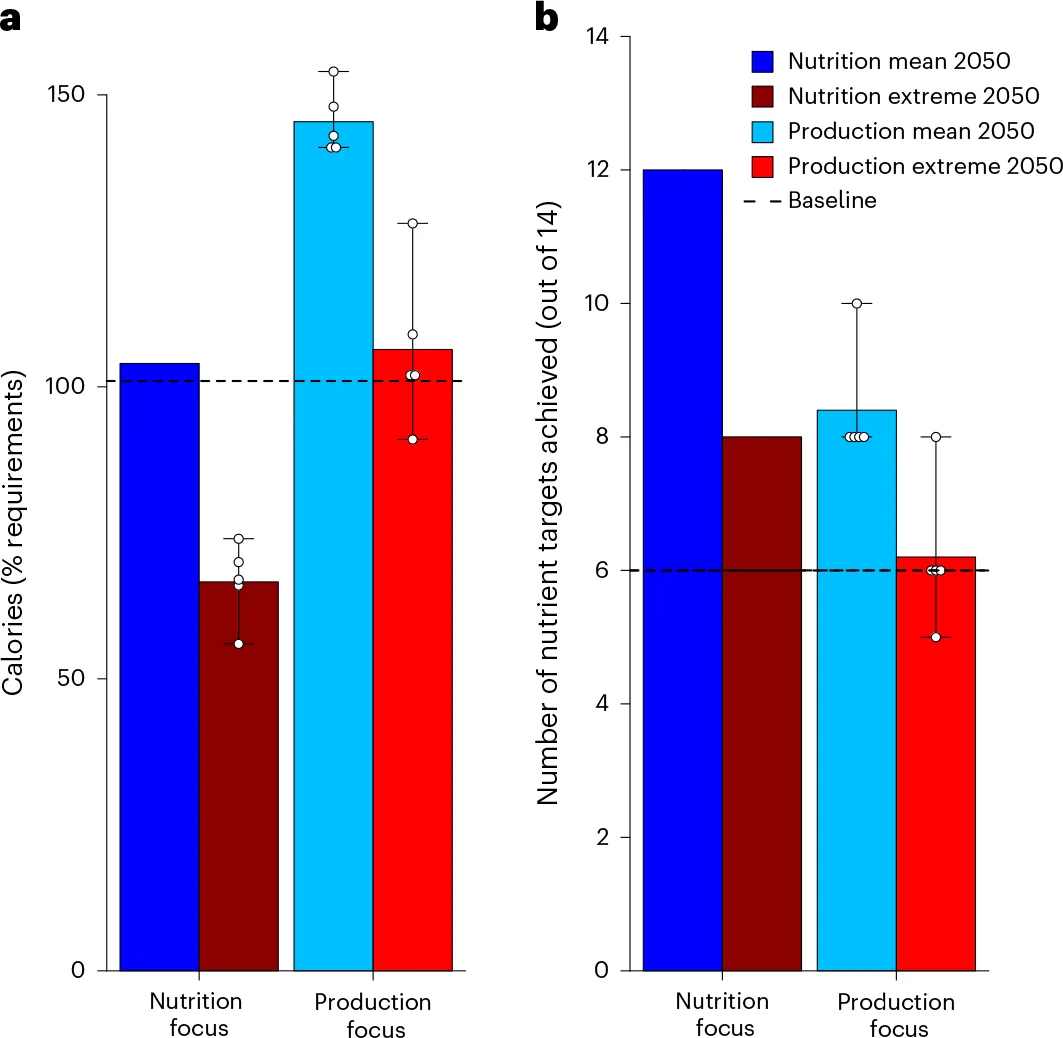
Summary of calorie and nutrient supplies relative to requirements by mid-century (2040–2060). **a**, Percentage of average calorie requirements. **b**, Number of macro- and micronutrient requirements achieved based on RNI (which is intake that would meet nutrient requirements for 97.5% of the population). Results are presented for an average future year (‘mean’) and extreme future year (‘extreme’, which shows the simultaneous largest future production shocks for each crop) for each scenario. The horizontal dashed line represents the baseline (1990–2010). Bars show mean results across five climate models, with error bars representing the range across climate models. No error bar indicates that all climate models have the same result. In **b**, if a macro- or micronutrient falls below 100% of requirements, it is considered not achieved. Source data

The nutrition focus scenario delivers required population-level calories and most (but not all; Methods) macro- and micronutrient requirements in average mid-century years (Fig. 1). This contrasts with extreme years (see Supplementary Section 2 for more details), where the nutrition focus scenario is not sufficient to avoid inadequate supplies of either calories or nutrients, although more requirements are met than in the production focus scenario, and typically nutrient supplies are above the baseline (Supplementary Table 1). Prioritizing nutrient-dense crops (for example, green vegetables, pulses and fruits) results in greater nutrient supplies than the production focus scenario, where a continued dominance of maize could link nutrient improvements to calorie overconsumption.

With the crops prioritized in the production focus scenario, imports would have to increase to achieve nutrition security in both average and extreme future years. Vegetable imports, in particular, would need to increase substantially to meet nutrient requirements. These are costly and would require infrastructural improvements such as supply chain cold storage to minimize losses. It would also be inherently risky, particularly in extreme future years, when regional production could also decline^20^.

For the nutrition focus scenario to also fulfil calorie and nutrient requirements in extreme future years, agricultural land could be further expanded, crop yields increased or imports increased. While these major policy levers are in theory available across SSA, country-level analysis is needed to support specific policy recommendations. We explore these options in turn for Zambia—assessing gaps between current policies and requirements for nutrition security in the most extreme future years.

**Fig. 2:**
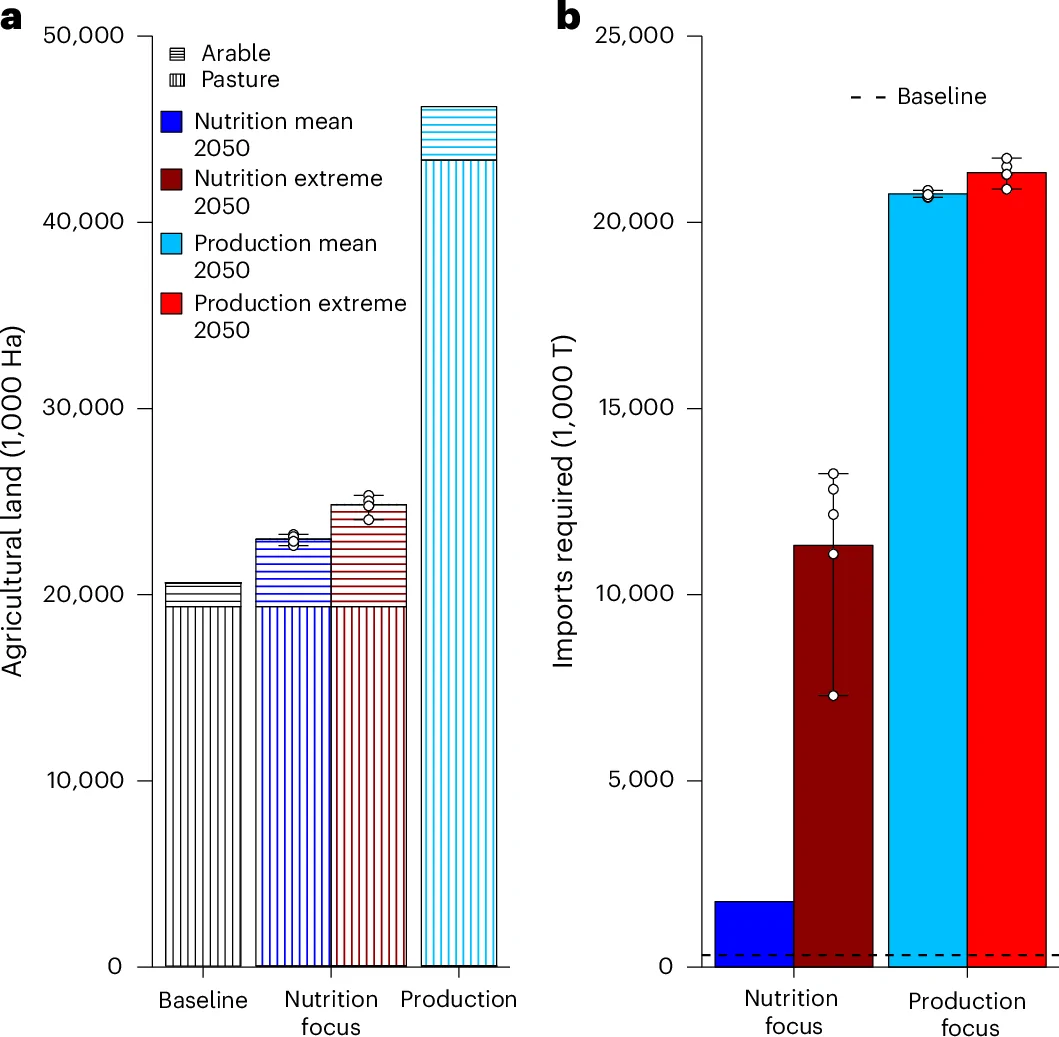
Summary of agricultural land and imports by mid-century (2040–2060). **a**, Agricultural land used. **b**, Imports required to achieve nutrition security. Baseline is 1990–2010. Results are presented for an average future year (‘mean’) and extreme future year (‘extreme’, which shows the simultaneous largest future production shocks for each crop) for each scenario. Bars show mean results across five climate models, with error bars representing the range across climate models. No error bar indicates that all climate models have the same result. For the nutrition focus scenario in **a**, the land use represents the amount required to meet production targets in average future climate, and extreme future years. For the production focus scenario in **a**, the land use is the same for both mean climate and extreme climate. The horizontal dashed line in **b** shows baseline food imports. Source data

Turning to the first of these levers, the arable land required to achieve nutrient requirements in extreme future years in the nutrition focus scenario is substantial, ranging from a 268% to 370% increase relative to the baseline of 1.3 million hectares (Fig. 2). This would increase cropland to potentially over 6 million hectares. The land expansion required is contingent on the crop yields achieved in 2050; if yield trends are half of those applied in the nutrition focus scenario, as high as 7 million hectares could be required. Zambia has more potentially available land than many other SSA countries, although expansion to this extent could require encroachment on densely populated or forested land^21^. Expanding non-staple crops, such as fruits and vegetables, could additionally require high levels of agricultural management. The Zambian Government’s Comprehensive Agriculture Transformation Support Programme (CATSP^15^) refers to ‘inadequate land under cultivation’ as a constraint on total production. In 2004, the Zambian government identified areas of 100,000 ha for agricultural expansion in each of the nine provinces. Modelling results suggest that, while agricultural expansion could provide sufficient food without encroaching on protected land, it would require over twice the expansion targeted by CATSP.

The second major lever is crop yields, which would have to approximately triple to hit production targets in extreme years in the nutrition focus scenario without the aforementioned cropland expansion. This is more than the approximate doubling of yields in the nutrition focus scenario, where historical yield trends (of 83% on average across crops, and 192% for maize) are assumed to continue. These increases are agronomically possible for SSA, with maize yields currently 2 t ha^−1^, but would require substantial investment in agricultural inputs, markets and institutions^22,23^. Such investment will be critical in all SSA countries, particularly so in countries with less land available for expansion.

Zambia has ambitious targets for increasing yields. CATSP includes 2027 targets of 4 t ha^−1^ for maize and 3.2 t ha^−1^ for soybean. The National Agricultural Policy^24^ advocates climate-smart agriculture and improved access to agricultural inputs. Our analysis suggests that policies should focus on commodity-specific targets based on nutritional requirements. This analysis highlights that improving the productivity of neglected and underutilized species such as millet and traditional green vegetables remains an adaptation gap that can contribute to climate resilience and improved nutrition^25^.

In Zambia’s National Agricultural Policy^24^, the rationale for increased production is largely around producing surplus for export. The Zambian National Trade Policy^26^ is heavily oriented towards exports and there is no reference to the importation of food. National nutrition and adaptation plans similarly omit food imports^16,27^, highlighting a critical policy gap. Given unprecedented required production increases in extreme years, imports could be required to fulfil short-term nutritional requirements (Fig. 2). Currently, Zambia imports approximately 5% of its domestic food supply, and 64% of these imports are maize and wheat; in the future, imports of a greater variety of foods (primarily vegetables) would be needed to meet nutrient requirements. Early warning systems have potential for identifying climate extremes and informing climate-resilient trade networks. Countries in SSA can identify what commodities are needed to fulfil key nutrient gaps in extreme years, and where these could come from. The challenge, however, is the costly nature of the imported foods and associated infrastructure required. Providing enough diverse cropland to fulfil country-level requirements should therefore be the priority given the challenges and risks associated with relying on food imports for nutrition security. Complementary strategies include increasing the capacity for storing perishable foods, transportation infrastructure and reducing post-harvest losses.

The major policy options discussed above are not straightforward to achieve. Tolerance to drought and heat stress has long been a focus in agronomy and crop breeding, but nutrition needs to be integrated into these plans. This would require strengthening agricultural research capacity in African institutions—even more so when concerned with neglected and underutilized crops, which are currently underresearched globally.

Food system resilience also goes beyond food supply measures. If adaptive policies are to truly bolster enduring resilience, they should go beyond merely technical approaches and address the broader social context in which they are implemented. This includes considering the conflict potential of climate-induced nutrition insecurity^28,29^, avoiding siloed thinking across government departments and policies that lead to the most vulnerable being further disadvantaged. Gender and social equity frameworks can also help vulnerable farmers benefit from adaptive policy interventions^30,31^ and equitable access to food. Land tenure security and social safeguards for vulnerable groups are mentioned in CATSP, although considering more comprehensive, conflict and equity-sensitive frameworks would help ensure nutrition security for the entire population. Indeed, without such frameworks, adaptive interventions risk adverse effects on marginalized populations without adequate consideration of vulnerability contexts^32^. More than anything, these policies need to integrate nutrition as a central pillar of agricultural policy, rather than a continued focus on improving the production of staple crops^33^.

## Methods

iFEED is a modelling framework used to calculate changes to crop yields, land use and food production and ultimately to assess nutrition security in 2050. In this analysis, we define nutrition security as adequate calorie and nutrient supplies to meet dietary requirements at a population level. For most nutrients, this is based on the Reference Nutrient Intake (RNI), which is the amount that is sufficient to ensure that the needs of 97.5% of individuals in the population are met. While RNI is based on individual requirements, a weighted average requirement was calculated based on population demographics and was used as this provides a benchmark for whether the national supply is theoretically sufficient. We acknowledge that this does not guarantee nutrition security at the individual level, as actual adequacy is contingent upon equitable food distribution, affordability and access, which are not measured in this supply-side analysis.

The iFEED modelling framework assesses nutrition security given changes to land use, food production and international trade. iFEED has been previously described^17^, and similar data and methods have been applied for Zambia^2^. Here, we describe our approach to assess future changes to land use, yields, food production and nutrition security for both average and extreme future years. A comparison of iFEED with alternative integrated assessment models for assessing future food systems is provided in Supplementary Section 3.

We first simulate climate change impacts on crop yields using the General Large Area Model for annual crops (GLAM^34–36^). GLAM is run on a 0.5° grid using a subset of bias-corrected climate data for Africa^19^ (Supplementary Informaton section 2) and soil data from the Harmonized World Soil Database (HWSD) version 1.2 (ref. ^37^). The soil input parameters required for crop modelling were the drained lower limit, drained upper limit and saturation limit. Percentage values of sand and clay, aggregated to the 0.5° grid, were used to calculate these hydrological parameters^38^. We compared HWSD versions 1.2 and 2 and found minimal difference in crop model results when driven by the two datasets (Supplementary Fig. 3). The crops modelled were maize, soybean, potato and groundnut. Simulations for these crops underpinned projections for the full range of crops grown in Zambia. Historical yield data^39^ were altered in future to represent climate change impacts using average projected changes from soybean, groundnut and potato for C3 crops, and using maize climate change projections for C4 crops. Simulations were conducted using a historical period around the year 2000 (1990–2010), and a future period centred on 2050 (2040–2060). Simulations include autonomous adaptation to climate change by allowing planting dates to change in response to climate and altering crop varieties to mitigate growing season reduction due to warming. Planting windows and crop varieties were selected based on the combination that returned the highest yield in each year (using a rolling 21-year average), with crop varieties defined using different maturity classes. Both scenarios applied the same technology trend to increase crop yields by 2050; for each crop, continuation of historical trends as seen in Food and Agriculture Organization of the United Nations Statistics Division (FAOSTAT^39^) yield data from 1960–2010 was applied, as in previous analyses^2,17^.

Baseline and future simulations assumed irrigated conditions according to Monthly Irrigated and Rainfed Crop Areas (MIRCA) data^40^. Irrigation grid cells were determined using a majority grid cell approach using the MIRCA data. If more than 50% of growing area in a grid cell was irrigated, fully irrigated (that is, no water stress) simulations were used in that grid cell.

We then calculate changes to agricultural land using iFEED land use allocation routines that maximize production on available land as described in the scenario land use allocation section below. Crop production changes to 2050 are then calculated using land use and yield projections. Livestock production changes are calculated using projected changes to livestock pasture, crop residues and crop production used as livestock feed, and assume historical relationships between livestock feed and livestock meat and dairy production remain the same by 2050^41^. This assumes that future livestock feed ratios and livestock productivity remain the same to 2050, agnostic to any changes to herd structures and production systems. For more details and implications of the livestock modelling, see Supplementary Section 1.

We use FAOSTAT^39^ Food Balance Sheet (FBS) data, along with crop nutrient content data^33^ and United Nations medium variant population projections, to assess changes to population-level nutrition security. In this analysis, we assume that, for each food commodity, international imports and exports remain in the same proportion to domestic production as in the baseline. The FBS data provide an estimate of the supply of food commodities based on domestic production, imports and exports, including stock variation of each commodity. In iFEED, the supply of energy and micronutrients are calculated as follows. FBS food commodities are converted to food as eaten, adjusting for unavoidable waste (such as inedible peel and bones) and household waste (uneaten edible food), as well as adjusting for losses from storage and transportation accounted for in the FBS, and food used for livestock feed, seed and processing. These food uses are assumed to continue in the same proportion to baseline domestic supply quantity in the future, that is, if 10% of food is lost in storage in the baseline, 10% is assumed lost in 2050. The food commodities are disaggregated into food items and matched to foods in a region-specific food composition table, which provides an estimate of calories, protein, fat, carbohydrate, saturated fat, fibre, calcium, zinc, iron, vitamin C, thiamin, riboflavin, niacin, folate and vitamin B6 per 100 g for each commodity group. Each food item is then weighted to represent the quantity of each food eaten at a country level, before being aggregated back to food commodity groups. We assume no changes to the weightings of foods within each food item between baseline and future for this calculation. Adequate nutrition requirements are set according to World Health Organization recommendations. The population-level nutrient requirements are adjusted for projected demographic changes (population size, age, sex and fertility rates) based on medium-variant United Nations projections to 2050.

In this analysis, we use iFEED to explore two contrasting scenarios that offer potential pathways towards nutrition security for Zambia given climate extremes: nutrition focus and production focus. The next two sections describe the methods used in the scenarios, and our methods to assess climate extremes, respectively.

### Scenario land use allocation

Both scenarios assume crop area expansion from 2000 to 2050 to different extents. This study prioritizes a yield-based approach to land use allocation that is focused on biophysical potential rather than complex economic modelling. We avoid uncertainties associated with markets, income, food prices and food demand changes to 2050 to determine agricultural land use. Optimization of land use is based on placing crops where they have the highest yield until area requirements are met, with the crops grown specified by total areas nationally and varying in each scenario as detailed below. Crop diversity at the level of each grid cell is determined by a parameter that specifies the maximum fraction of each crop that can be placed in each grid cell. This parameter was specified to be the same for both scenarios, and to be as low as possible while still allowing national crop targets to be met for all climate models.

The production focus scenario maximizes the crop production on available land by optimizing the placement of crops based on simulated yields in the future period, without considering nutritional requirements. The scenario assumes a maximum possible increase in agricultural land, which was determined using Land-Use Harmonization II data^42^, and assumes all land was available for agricultural expansion if not forested, urban or protected according to The World Database on Protected Areas^43^. The total areas of each crop nationally are kept in the same proportion in the future as in the baseline; using the total arable area, and these proportions, future crop area targets are calculated. To maximize production, crops were allocated to grid cells using a ‘highest-yield-first’ priority ranking. In this approach, land was allocated to the highest-yielding crop–cell combination first, continuing iteratively until either the physical land within a grid cell was exhausted or the precalculated national area target for a specific crop was reached.

The nutrition focus scenario is designed to improve nutrition security using changes to crop and livestock production to meet nutrient requirements by mid-century, minimizing cropland expansion, and without expanding pasture for livestock, to minimize the negative environmental impacts associated with land use expansion. We first calculate the quantities of crop and livestock production required to ensure nutrition security per person, minimizing changes to current diets. This step uses linear programming to calculate per capita food commodity (FAOSTAT FBS data) requirements that achieve nutrition security. We allow changes to food commodities derived from only the crop and non-ruminant livestock (primarily pig and poultry) commodities produced in Zambia. The crop commodities included in the linear programming are ‘Wheat and products’, ‘Maize and products’, ‘Millet and products’, ‘Sorghum and products’, ‘Sugar cane’, ‘Barley and products’, ‘Cassava and products’, ‘Groundnuts (Shelled Eq)’, ‘Onions’, ‘Potatoes and products’, ‘Rice (Milled Equivalent)’, ‘Cottonseed’, ‘Sunflower seed’, ‘Sweet potatoes’, ‘Tomatoes and products’, ‘Vegetables, Other’, ‘Bananas’, ‘Fruits, Other’, ‘Oranges, Mandarines’, ‘Pulses, Other and products’ and ‘Soyabeans’. In most SSA production systems, ruminants rely on natural pastures and crop residues, which account for the majority of feed despite recent increases in fodder crop intake^44,45^. Therefore, no change to ruminants per person was permitted in the linear programming, as pasture does not expand in this scenario. The linear programming minimized the total amount of change from current food supplies, with nutrient requirements as constraints. A maximum possible decrease of 50% was allowed for each commodity, with this constraint increased in increments of 5% until a solution was reached; this ensured that the solution to meet all nutrient requirements minimized changes from current diets. Using the per capita, per commodity amounts from the linear programming, we then calculate the required changes in domestic crop and livestock production needed to support these dietary requirements by 2050 given population growth. We lastly take these crop production amounts and allocate land use to hit these targets. We minimize the cropland needed to support this food production by placing crops in grid cells that have the highest yields until production targets are achieved. This optimization does not consider food prices given future uncertainties in how these could change; instead, we minimize changes to current diets to meet requirements while minimizing potentially culturally insensitive changes, and minimizing any changes to affordability. Explicitly accounting for affordability in this optimization could be explored in future analysis, although optimizing to minimize costs would probably result in larger changes from current diets to meet requirements.

The linear programming fixes as constant the per person amounts of FAOSTAT FBS commodities that are not grown domestically. It also fixes as constant commodities that we do not simulate owing to a lack of historical evaluation data, primarily fish products and the ‘Meat, Other’ commodity. In total, 55 commodities are eaten, processed into food or used as livestock feed in the FBS. Thirteen of these commodities are not included in the linear programming owing to lack of data or because they are not growing domestically. Absolute amounts of these commodities therefore remain the same in the nutrition focus 2050 scenario, resulting in per person reductions in these commodities. With business-as-usual imports accounted for, this results in deficient calcium and inadequate fat at the population level; an insufficient calorie, zinc and fibre supply can also occur if assuming no access to food imports in the future. An alternative would have been to compensate in the linear programming for the per capita reductions of these commodities; this would involve introducing further changes to current food supplies, in that per capita amounts of these commodities would reduce and be compensated by others. We favoured the simpler assumption of not changing the non-simulated commodities.

### Climate extremes

We drive the iFEED modelling framework with Coupled Model Intercomparison Project Phase 5 (CMIP5) climate projections^19^. These data were bias-corrected to more accurately represent the African climate using WFDEI data (WATCH Forcing Data methodology applied to ERA-Interim data) and the cumulative distribution function transform (CDF-t) method.

A subset of the bias-corrected CMIP5 climate data was used to assess nutrition security in an average future year and an extreme future year that represents the simultaneous largest possible shocks to the production of each crop from drought and high temperatures. We focus on RCP8.5, and this subset, to explore the plausible worst-case production shocks in this crop-climate ensemble by mid-century and how food systems might adapt to these shocks to ensure food system resilience. We assume the expanded growing areas as used in the production focus scenario to calculate crop production to determine the subset. The crop projections used to select the subset are maize, soybean, groundnut and potato—the four crops that are simulated using GLAM in this analysis that form the basis of wider crop–climate projections as in previous iFEED analysis^2,17^. The crop yield simulations in both production focus and nutrition focus scenarios represent adaptation to climate change through changes to planting dates, and changes to crop varieties to compensate for warming-induced reduction in the crop growing season^2,17^. GLAM does not simulate flood impacts; therefore, production shocks are driven by low rainfall and high temperatures. Crop yield shocks are projected to approximately double in frequency without adaptation to climate change with RCP8.5^2,46^.

A subset of five climate models was selected for RCP8.5 by calculating the largest food production shocks (the largest difference in production between the average and worst future year, normalized by future production standard deviation; other ranking methods were tested, such as inclucing coefficient of variation, and found to result in similar climate model rankings), ranking the worst models for each crop and selecting the five models that gave the aggregate lowest ranking of production years across all modelled crops. We then calculated food production in an average future year for this subset, and an extreme future year. The extreme future year results were obtained by aggregating the simultaneous largest possible shocks to the production of each crop.

Compared with the full bias-corrected CMIP5 ensemble and the representative subset of five models used in previous iFEED analysis^2^, the subset used in this analysis has comparable mean changes in production:

- Maize: whole ensemble −15.8%, previous subset −17.7%, current subset −16.4%.
- Soybean: whole ensemble −22.4%, previous subset −24.4%, current subset −21.5%.
- Groundnut: whole ensemble −11.8%, previous subset −13.2%, current subset −11.4%.
- Potato: whole ensemble −16.3%, previous subset −16.9%, current subset −17.2%.

The temperature and precipitation changes in the iFEED CMIP5 subset sit within the broader range across the ensembles of CMIP5 and CMIP6 models (Supplementary Fig. 2).

### Reporting summary

Further information on research design is available in the Nature Portfolio Reporting Summary linked to this article.

## Supplementary information

Supplementary Information Supplementary discussion on livestock and nutrition implications, climate shocks and integrated assessment models; Supplementary Table 1 showing changes in nutrients relative to the baseline; Supplementary Fig. 1 showing all macro- and micronutrient supplies relative to requirements; Supplementary Fig. 2 showing climate model ensemble information; and Supplementary Fig. 3 showing sensitivity analysis of different soil data.

Reporting Summary

## Source data

Source Data Fig. 1 Source data for Fig. 1.

Source Data Fig. 2 Source data for Fig. 2.

## Data Availability

The data generated and analysed during this study are available via Zenodo at 10.5281/zenodo.18760105 (ref. ^47^). Input data used in this study are from publicly available sources and referenced in ref. ^17^. In summary, these consist of the following: the CDF-t bias-corrected CMIP5 data over Africa, available at http://amma2050.ipsl.upmc.fr/ (to access the data, users must contact the lead author at moflod@locean-ipsl.upmc.fr.); FAOSTAT yield and area and Food Balance Sheet data, available at https://www.fao.org/faostat/; soil data from the Regridded Harmonized World Soil Database v 1.2, available at https://www.fao.org/soils-portal/data-hub/soil-maps-and-databases/harmonized-world-soil-database-v12/en/; and gridded area data from LUH2 (https://luh.umd.edu/) and WDPA (https://www.protectedplanet.net/en/thematic-areas/wdpa?tab=WDPA). Source data are provided with this paper.

## Code availability

Code for the food production calculations and nutrition security analysis is available via Zenodo at 10.5281/zenodo.18760105 (ref. ^47^). The General Large Area Model for annual crops (GLAM) was used for the crop yield simulations. An older version of this model is available at https://licensing.leeds.ac.uk/product/general-large-area-model-for-annual-crops-glam. The version (version number 79e1615) used for the simulations in this Brief Communication is available upon reasonable request. Notable updates are new crop routines for potato and soybean, and the ability to simulate biomass from radiation use efficiency (as opposed to only transpiration efficiency; see ref. ^35^ for details).
