# Supplementary material for: Adaptive policies balancing trade, productivity and cropland increases can support Zambia’s nutrition security under future climate shocks
Source: Nat Food. 2026 Jul 28;7(8):756–62. doi: 10.1038/s43016-026-01405-1 (PMC13489942; doi:10.1038/s43016-026-01405-1)
Supplement: Supplementary file 1 [file 43016_2026_1405_MOESM1_ESM.pdf]

nature food

Supplementary information https://doi.org/10.1038/s43016-026-01405-1

Adaptive policies balancing trade, productivity and cropland increases can support Zambia's nutrition security under future climate shocks

In the format provided by the authors and unedited

1 Livestock changes and nutrition security implications

Livestock production changes are calculated using projected changes to livestock pasture, crop residues and crop production used as livestock feed. Continuation of historical relationships between livestock feed and livestock meat and dairy production is assumed by 2050. These relationships are calculated using data [1] in the following categories: bovine meat, bovine milk, sheep and goat meat, sheep and goat milk, pig meat, poultry meat and eggs.

More land is used in the production focus scenario compared to the nutrition focus scenario, mostly due to pasture expansion. In the production focus scenario, increases in meat and dairy production in an average future year are greater than 300% in all cases and sometimes more than 400%. For context, meat production (including pigs, poultry, bovids, sheep, goats and other meats) in the baseline in Zambia is approximately 162,000 tonnes, and milk production is 72,000 tonnes; and by comparison, South Africa's bovine meat production alone is 953,000 tonnes, and milk production is over 3 million tonnes. The nutrition focus scenario shows smaller increases in meat and dairy production, although these are still substantial, with increases larger than 200% in most cases. Because the Zambian population is projected to increase by 267% by mid-century, these changes in livestock production do not translate to significant changes in nutrition security. Some increase in emissions could be regarded as tolerable to achieve nutrition security [2, 3]. However, increasing livestock production further would require more land, and more emissions, than is necessary to fulfil nutrient requirements.

Several micronutrients supplies are inadequate due in extreme years (iron, zinc, fibre and niacin in the nutrition focus scenario for all climate models; folate, iron, zinc, riboflavin and niacin for at least one climate model in the production focus scenario), but typically still above the baseline (where vitamin A, folate, calcium, iron, zinc, fibre and riboflavin are all insufficient).

Some nutrient requirements are not met in the nutrition focus scenario, even in an average future year, with small inadequacies in the supply of fat and calcium (see the Methods section for the reasons why). There are excesses of livestock feed produced in this scenario that could be used to support additional livestock meat and dairy production and meet these requirements. Although fat and calcium could be alternatively sourced from nuts, pulses and

green vegetables, in SSA, and in particular among women (who have higher requirements than men) adequate supply of iron can be more difficult to achieve from diets lacking in meat and dairy [4]. In the production focus scenario, even in an average future year, supplies of fat and calcium are inadequate, with vitamin A and fibre also below requirements. There is also an over-supply of calories, with an average of 145% of per capita requirements. This would increase the risk of overconsumption of calories to fulfil nutrient requirements.

2 Production shocks

The impacts of extreme future years on crop production shocks, and therefore nutrition security, are substantial in both scenarios. The largest crop-specific shocks can result in production shortfalls of more than half compared to an average future year. In the production focus scenario, total crop production is 15-35% lower, depending on the climate model, in extreme future years than in an average future year. This situation is slightly worse (21-41%) in the nutrition focus scenario. When looking at maize alone, this difference is starker, with production losses of 26% in the production focus and 40% in the nutrition focus scenario.

Both scenarios show a larger range across climate models in production values, and therefore nutrient supplies, for extreme years compared to mean years (Figure SI1). Climate models show greater uncertainty in extremes compared to means a result consistent with previous studies [5].

Crop production in Zambia is predominantly rainfed but there is potential to expand small-scale irrigation on existing cultivated land [6]. Irrigation can reduce maize yield shocks by over 40% [2]. Irrigation also facilitates additional cropping seasons when rainfall would otherwise be limiting, particularly for short season crops such as vegetables (which are critical in the nutrition focus scenario). The Zambian government has proposed interventions in response to the 2023/2024 drought event, including improving investment in irrigation technologies and water harvesting, as well as improving financial support for farmers through the Constituency Development Fund for irrigation and climate resilience, the Citizens Economic Empowerment Commission and the Sustainable Agricultural Financing Facility. Our results indicate that increased investment in irrigation is necessary but not sufficient to mitigate climate shocks and ensure nutrition security.

3 iFEED and Integrated Assessment Models

Various models of differing complexity are used to evaluate how climate change affects future food systems. Integrated Assessment Models (IAMs) link biophysical and socio-economic systems through economic equilibrium theories - such as partial equilibrium models - which endogenize trade and production decisions by balancing supply, demand, and prices. While these frameworks can capture how fluctuating export prices influence farmer behaviour or how imports fill nutritional gaps, they rely heavily on assumptions regarding market stability, changing food prices, and dietary trends (e.g., the correlation between wealth and meat consumption, or that supplies will shift to meet changing demands). These trends are uncertain, for example due to the simultaneous "westernisation" of diets in sub-Saharan Africa and the shift toward plant-based proteins in the global north. IAM assumptions will perhaps also more readily break down in contexts with weaker infrastructure and a less certain link between supply and demand. IAM outputs can additionally be difficult to interpret with confidence given the complexity of interrelated modelling processes.

In contrast, our study prioritises a simpler, more transparent yield-based optimisation approach. By focusing on biophysical potential rather than endogenising economic drivers, we avoid the compounding uncertainties of markets and to what extent dietary demands and prices will change. Instead, we rely on scenario analysis and expert interpretation to evaluate socio-economic processes. This allows for a more transparent assessment of biophysical constraints without the "black box" complexity of equilibrium-driven simulations, providing a baseline of what is physically attainable before economic factors are considered. We believe that both IAMs and iFEED have strengths and can provide complementary information on the future of the food system. A broader discussion of the limitations of iFEED can be found in previous work [3, 7].

97 # 4 Supplementary Tables and Figures

Table 1 Baseline (1990 to 2010) nutrient requirements (% achieved) and percentage changes for each scenario by mid-century (2040-2060) relative to baseline. Figures are mean, min and max across climate models. Blue text indicates all climate models are greater than the baseline; red text indicates all climate models are lower than the baseline; amber text indicates some climate models are lower and some higher than the baseline.

	Baseline	Nutri.Mean	Nutri.Extreme	Prod.Mean	Prod.Extreme
**Dietary Energy**	101	3 (3, 3)	-34 (-44, -27)	45 (40, 53)	**6 (-9, 27)**
**Protein**	159	32 (32, 32)	-29 (-46, -16)	56 (47, 69)	**0 (-23, 30)**
**Fat**	64	-8 (-8, -8)	-24 (-29, -20)	-3 (-3, -2)	-4 (-9, -2)
**Saturated Fat**	28	-4 (-4, -4)	-10 (-11, -9)	-8 (-8, -8)	-8 (-10, -7)
**Vitamin C**	210	513 (513, 513)	278 (210, 389)	79 (62, 93)	**-10 (-42, 45)**
**Vitamin A**	52	150 (150, 150)	96 (81, 121)	16 (13, 19)	**-1 (-8, 10)**
**Folate**	69	117 (117, 117)	59 (45, 80)	27 (24, 33)	**1 (-10, 16)**
**Calcium**	22	64 (64, 64)	37 (31, 47)	7 (5, 8)	**-1 (-4, 3)**
**Iron**	65	58 (58, 58)	17 (10, 27)	30 (27, 36)	**5 (-5, 18)**
**Zinc**	85	19 (19, 19)	-16 (-25, -9)	47 (42, 54)	**11 (-3, 31)**
**Dietary Fibre**	48	61 (61, 61)	25 (18, 35)	18 (16, 22)	**0 (-7, 10)**
**Thiamin**	176	48 (48, 48)	-30 (-53, -12)	91 (82, 108)	**19 (-10, 57)**
**Riboflavin**	90	132 (132, 132)	64 (51, 85)	38 (34, 46)	**5 (-8, 24)**
**Niacin**	106	25 (25, 25)	-17 (-26, -9)	42 (37, 51)	**3 (-13, 25)**
**Vitamin B6**	181	32 (32, 32)	-41 (-62, -25)	96 (86, 113)	**20 (-10, 61)**

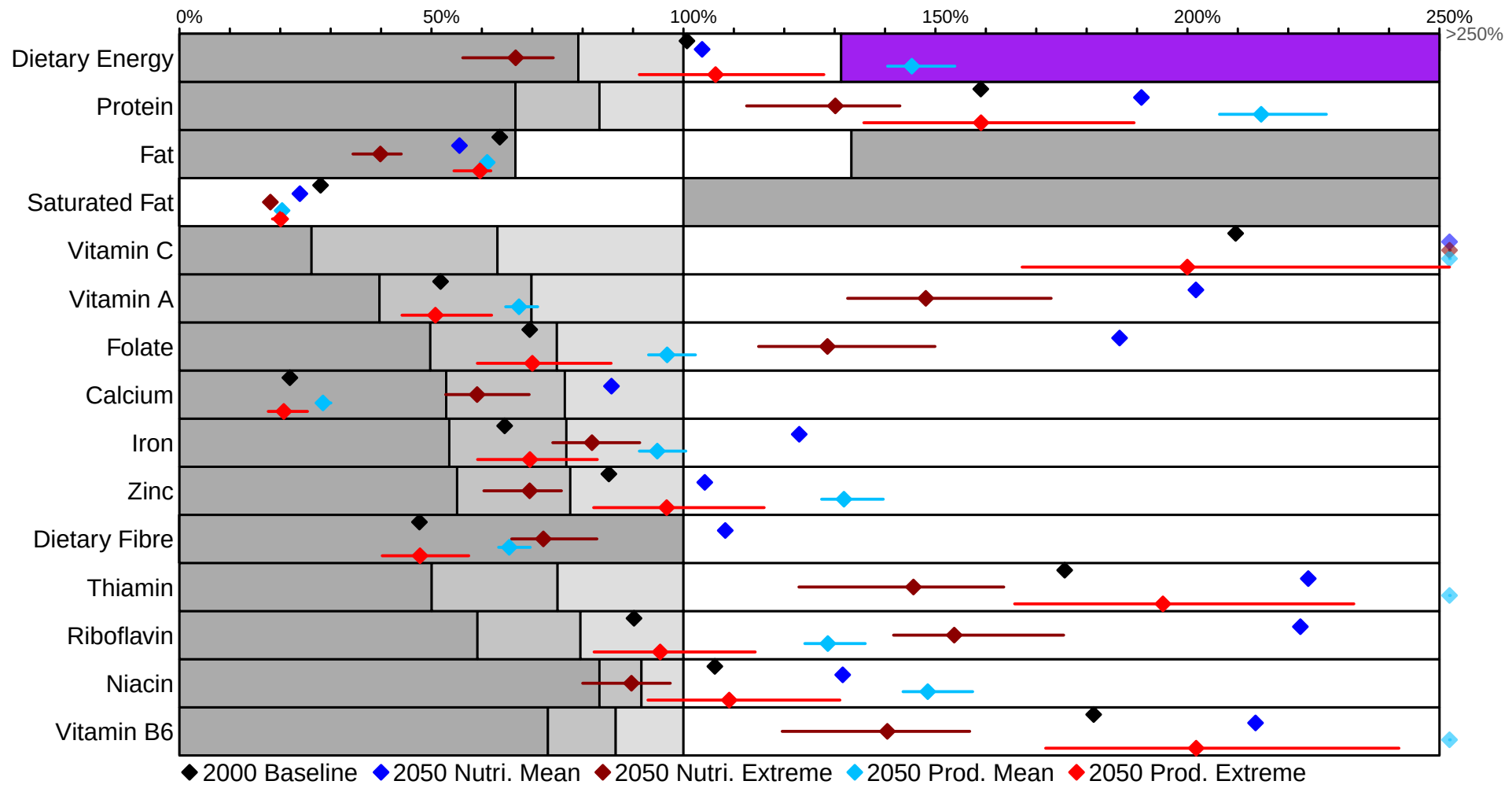

Fig. 1 Per capita nutrient supplies relative to population requirements (100%) for each scenario Black diamonds indicate baseline (2000) per capita nutrient levels. The four coloured diamonds indicate projected outcomes in 2050 under different scenarios, and the range shown for each scenario is across the five climate models. Diamonds appearing to the right of the figure are associated with greater than 250% of requirements. White areas indicate where per capita nutrient requirements are met. Grey areas indicate that requirements are not achieved, with progressively darker grey areas representing increasing levels of inadequacy at the population level. For all nutrients other than calories (dietary energy) and fat and protein, the first threshold represents the Lower Reference Nutrient Intake; the second, the Estimated Average Requirement; the third, the Reference Nutrient Intake (principal target). For fat and protein, thresholds correspond to minimum, minmax midpoint and maximum recommended intakes, respectively. For calories (dietary energy), the respective thresholds are minimum, average and maximum dietary energy requirements. The purple area indicates where calories are greater than requirements. Vitamin A is measured in retinol activity equivalents.

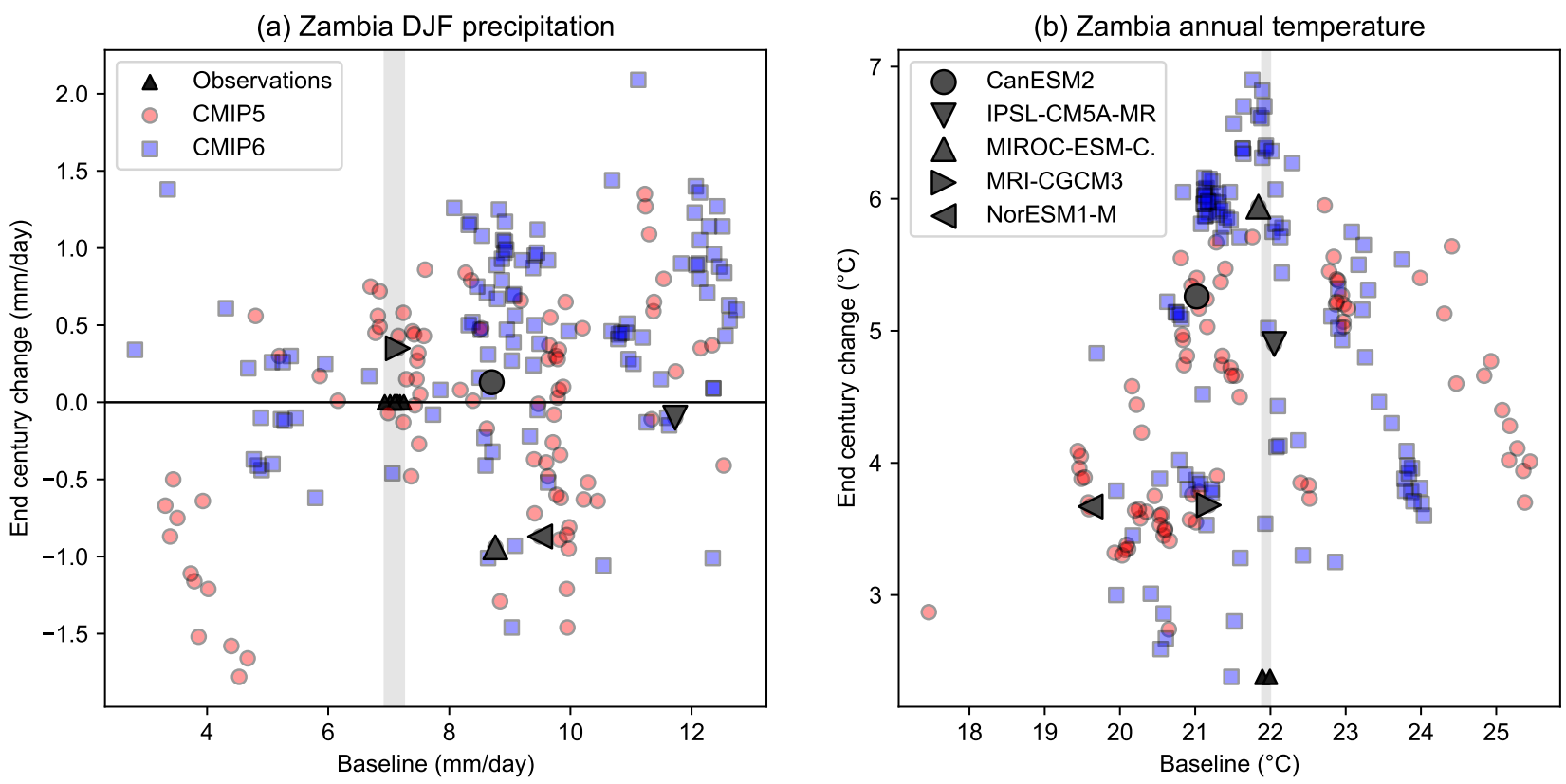

Fig. 2 Summary of precipitation and temperature climate input data and bias compared to observations. Modelled historic (1985-2014) and projected change in (a) mean December-January-February precipitation (mm/day) and (b) mean annual temperature (°C) over Zambia, showing all CMIP5 and CMIP6 models and ensemble members with monthly data available. iFEED models are shown by larger makers. Observations for the historic period are shown by black markers with maximum and minimum shown by the vertical grey band. For precipitation, observations used were: CHIRPS [8], CRU [9], GPCC [10], GPCP [11], REGEN-ALL [12], MSWEP [13], PERSIANN-CDR [14] and TAMSAT [15]. For temperature, observations used were: ERA5 [16] and Berkeley Earth [17].

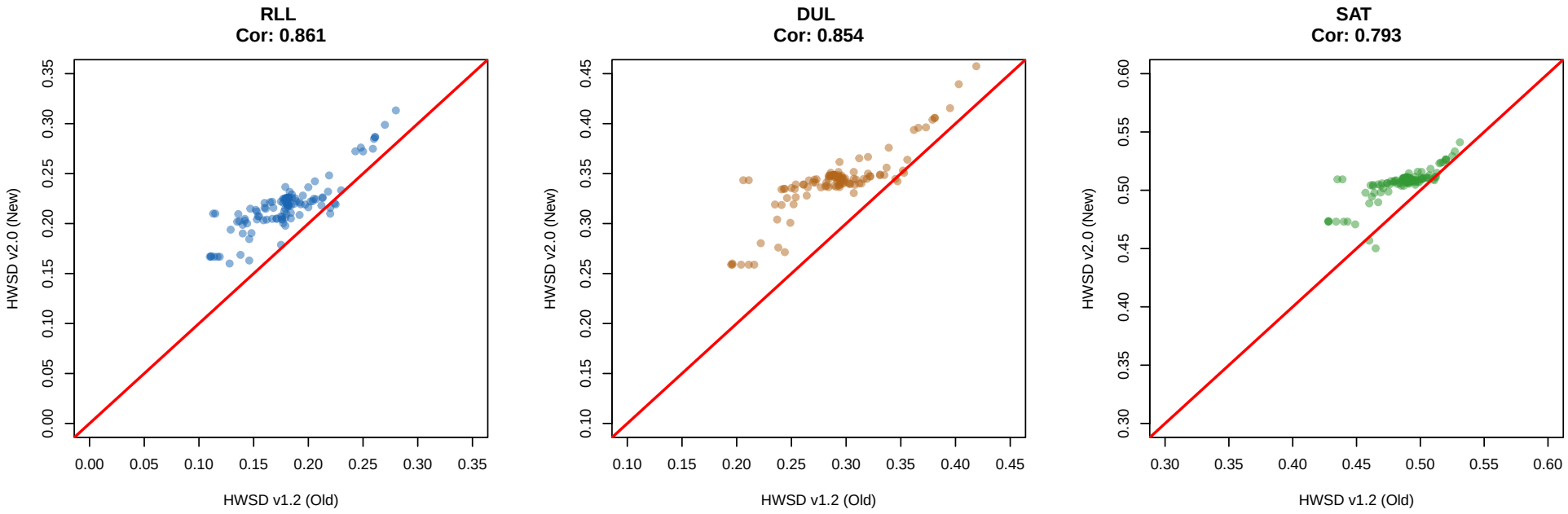

Fig. 3 Comparison of soil hydrological parameters in GLAM using Harmonized World Soil Database (HWSD) v1.2 and v2. Using HWSD v2 data, soil texture data (sand and clay percentages) were extracted for the top five depth layers (0100 cm) and processed through pedotransfer functions to derive critical water retention limits as was previously done with the v1.2 data: the drained upper limit (DUL), lower limit (RLL) and saturation limit (SAT) of the soil. We then compare these values and conduct a sensitivity analysis in GLAM that compares yields using the same model configuration used to evaluate the model in the historical period. While highly correlated (R¿0.85 for RLL and DUL), HWSD v2 consistently estimates higher water retention limits (RLL, DUL, SAT) than v1.2 across Zambia. While the use of different soil data can contribute to significant changes in crop model outputs [18], in this case the differences in GLAM simulations driven by the two datasets were small (mean yields of 2608 kg/ha and standard deviation 305 kg/ha and with v1.2, mean yields of 2568 kg /ha and standard deviation 301 kg/ha).

References

[1] Herrero, M., Havlík, P., Valin, H., Notenbaert, A., Rufino, M.C., Thornton, P.K., Blümmel, M., Weiss, F., Grace, D., Obersteiner, M.: Biomass use, production, feed efficiencies, and greenhouse gas emissions from global livestock systems. Proceedings of the National Academy of Sciences **110**(52), 20888–20893 (2013)

[2] Arenas-Calle, L., Jennings, S., Challinor, A.: Agro-technology for climate-smart agriculture and resilience to climate extremes in Sub-Saharan Africa. Environmental Research: Food Systems (2024)

[3] Jennings, S., Challinor, A., Smith, P., Macdiarmid, J.I., Pope, E., Chapman, S., Bradshaw, C., Clark, H., Vetter, S., Fitton, N., *et al.*: Stakeholder-driven transformative adaptation is needed for climate-smart nutrition security in sub-saharan africa. Nature Food **5**, 1–11 (2024)

[4] Raiten, D.J., Allen, L.H., Slavin, J.L., Mitloehner, F.M., Thoma, G.J., Haggerty, P.A., Finley, J.W.: Understanding the intersection of climate/environmental change, health, agriculture, and improved nutrition: a case study on micronutrient nutrition and animal source foods. Current Developments in Nutrition **4**(7), 087 (2020)

[5] Lesk, C., Anderson, W., Rigden, A., Coast, O., Jägermeyr, J., McDermid, S., Davis, K.F., Konar, M.: Compound heat and moisture extreme impacts on global crop yields under climate change. Nature Reviews Earth & Environment **3**(12), 872–889 (2022)

[6] Xie, H., You, L., Wielgosz, B., Ringler, C.: Estimating the potential for expanding smallholder irrigation in Sub-Saharan Africa. Agricultural Water Management **131**, 183–193 (2014)

[7] Jennings, S.A., Challinor, A.J., Smith, P., Macdiarmid, J., Pope, E., Chapman, S., Bradshaw, C., Clark, H., Vetter, S., Fitton, N., King, R., Mwamakamba, S., Madzivhandila, T., Mashingaidze, I., Chomba, C., Nawiko, M., Nyhodo, B., Mazibuko, N., Yeki, P., Kuwali, P., Kambwiri, A., Kazi, V., Kiama, A., Songole, A., Coskeran, H., Quinn, C., Sallu, S., Dougill, A., Whitfield, S., Kunin, B., Meebelo, N., Jamali, A., Kantande, D., Makundi, P., Mbungu, W., Kayula, F., Walker, S., Zimba, S., Yamdeu,

J.H.G., Kapulu, N., Galdos, M.V., Eze, S., Tripathi, H., Sait, S.M., Kepinski, S., Likoya, E., Greathead, H., Smith, H.E., Mahop, M.T., Harwatt, H., Muzammil, M., Horgan, G., Benton, T.: A new integrated assessment framework for climate-smart nutrition security in sub-Saharan Africa: the integrated Future Estimator for Emissions and Diets (iFEED). Frontiers in Sustainable Food Systems (2022)

[8] Funk, C., Peterson, P., Landsfeld, M., Pedreros, D., Verdin, J., Shukla, S., Husak, G., Rowland, J., Harrison, L., Hoell, A., *et al.*: The climate hazards infrared precipitation with stationsa new environmental record for monitoring extremes. Scientific data **2**(1), 1–21 (2015)

[9] Harris, I., Osborn, T.J., Jones, P., Lister, D.: Version 4 of the CRU TS monthly high-resolution gridded multivariate climate dataset. Scientific data **7**(1), 109 (2020)

[10] Ziese, M., Rauthe-Schöch, A., Becker, A., Finger, P., Meyer-Christoffer, A., Schneider, U.: GPCC full data daily version 2018 at 1.0: Daily land-surface precipitation from rain-gauges built on GTS-based and historic data (2018)

[11] Adler, R.F., Huffman, G.J., Chang, A., Ferraro, R., Xie, P.-P., Janowiak, J., Rudolf, B., Schneider, U., Curtis, S., Bolvin, D., *et al.*: The version-2 global precipitation climatology project (GPCP) monthly precipitation analysis (1979–present). Journal of hydrometeorology **4**(6), 1147–1167 (2003)

[12] Contractor, S., Donat, M.G., Alexander, L.V., Ziese, M., Meyer-Christoffer, A., Schneider, U., Rustemeier, E., Becker, A., Durre, I., Vose, R.S.: Rainfall estimates on a gridded network (regen)–a global land-based gridded dataset of daily precipitation from 1950 to 2016. Hydrology and Earth System Sciences **24**(2), 919–943 (2020)

[13] Beck, H.E., Van Dijk, A.I., Levizzani, V., Schellekens, J., Miralles, D.G., Martens, B., De Roo, A.: MSWEP: 3-hourly 0.25 global gridded precipitation (1979–2015) by merging gauge, satellite, and reanalysis data. Hydrology and Earth System Sciences **21**(1), 589–615 (2017)

[14] Ashouri, H., Hsu, K.-L., Sorooshian, S., Braithwaite, D.K., Knapp, K.R.,

Cecil, L.D., Nelson, B.R., Prat, O.P.: PERSIANN-CDR: Daily precipitation climate data record from multisatellite observations for hydrological and climate studies. Bulletin of the American Meteorological Society **96**(1), 69–83 (2015)

[15] Maidment, R.I., Grimes, D., Black, E., Tarnavsky, E., Young, M., Greatrex, H., Allan, R.P., Stein, T., Nkonde, E., Senkunda, S., *et al.*: A new, long-term daily satellite-based rainfall dataset for operational monitoring in Africa. Scientific data **4**(1), 1–19 (2017)

[16] Hersbach, H., Bell, B., Berrisford, P., Hirahara, S., Horányi, A., Muñoz-Sabater, J., Nicolas, J., Peubey, C., Radu, R., Schepers, D., *et al.*: The era5 global reanalysis. Quarterly journal of the royal meteorological society **146**(730), 1999–2049 (2020)

[17] Rohde, R.A., Hausfather, Z.: The Berkeley Earth land/ocean temperature record. Earth System Science Data Discussions **2020**, 1–16 (2020)

[18] Folberth, C., Skalskỳ, R., Moltchanova, E., Balkovič, J., Azevedo, L.B., Obersteiner, M., Van Der Velde, M.: Uncertainty in soil data can outweigh climate impact signals in global crop yield simulations. Nature Communications **7** (2016)
