# Supplementary material for: Adaptive policies balancing trade, productivity and cropland increases can support Zambia’s nutrition security under future climate shocks
Source: Nat Food. 2026 Jul 28;7(8):756–62. doi: 10.1038/s43016-026-01405-1 (PMC13489942; doi:10.1038/s43016-026-01405-1)
Supplement: Supplementary file 2 [file 43016_2026_1405_MOESM2_ESM.pdf]

nature portfolio

Corresponding author(s): Stewart Jennings

Last updated by author(s): 23/06/2026

Reporting Summary

Nature Portfolio wishes to improve the reproducibility of the work that we publish. This form provides structure for consistency and transparency in reporting. For further information on Nature Portfolio policies, see our Editorial Policies and the Editorial Policy Checklist.

Statistics

For all statistical analyses, confirm that the following items are present in the figure legend, table legend, main text, or Methods section.

n/a	Confirmed	
☐	☒	The exact sample size (*n*) for each experimental group/condition, given as a discrete number and unit of measurement
☒	☐	A statement on whether measurements were taken from distinct samples or whether the same sample was measured repeatedly
☒	☐	The statistical test(s) used AND whether they are one- or two-sided<br>*Only common tests should be described solely by name; describe more complex techniques in the Methods section.*
☒	☐	A description of all covariates tested
☒	☐	A description of any assumptions or corrections, such as tests of normality and adjustment for multiple comparisons
☒	☐	A full description of the statistical parameters including central tendency (e.g. means) or other basic estimates (e.g. regression coefficient) AND variation (e.g. standard deviation) or associated estimates of uncertainty (e.g. confidence intervals)
☒	☐	For null hypothesis testing, the test statistic (e.g. *F*, *t*, *r*) with confidence intervals, effect sizes, degrees of freedom and *P* value noted<br>*Give P values as exact values whenever suitable.*
☒	☐	For Bayesian analysis, information on the choice of priors and Markov chain Monte Carlo settings
☒	☐	For hierarchical and complex designs, identification of the appropriate level for tests and full reporting of outcomes
☒	☐	Estimates of effect sizes (e.g. Cohen's *d*, Pearson's *r*), indicating how they were calculated

Our web collection on statistics for biologists contains articles on many of the points above.

Software and code

Policy information about availability of computer code

Data collection

No software was used for data collection.

Data analysis

Code for the food production calculations and nutrition security analysis are available in the Zenodo repository at https://doi.org/10.5281/zenodo.18760105

The General Large Area Model for annual crops (GLAM) was used for the crop yield simulations. An older version of this model is available online https://licensing.leeds.ac.uk/product/general-large-area-model-for-annual-crops-glam. The version used for the simulations in this paper is available upon reasonable request. Significant updates are new crop routines for potato and soybean, and the ability to simulate biomass from radiation use efficiency (as opposed to only transpiration efficiency; see this paper for details https://www.sciencedirect.com/science/article/pii/S0168192312002407).

The methods used have been previously fully described in Jennings et al. (2022), A New Integrated Assessment Framework for Climate-Smart Nutrition Security in sub-Saharan Africa: The Integrated Future Estimator for Emissions and Diets (iFEED). Front. Sustain. Food Syst., 22 July 2022. Sec. Climate-Smart Food Systems .
https://doi.org/10.3389/fsufs.2022.868189.

For manuscripts utilizing custom algorithms or software that are central to the research but not yet described in published literature, software must be made available to editors and reviewers. We strongly encourage code deposition in a community repository (e.g. GitHub). See the Nature Portfolio guidelines for submitting code & software for further information.

Data

Policy information about availability of data

All manuscripts must include a data availability statement. This statement should provide the following information, where applicable:

- Accession codes, unique identifiers, or web links for publicly available datasets
- A description of any restrictions on data availability
- For clinical datasets or third party data, please ensure that the statement adheres to our policy

The data generated and analyzed during this study are available in the Zenodo repository at https://doi.org/10.5281/zenodo.18760105

Source data for Figures 1 and 2 are provided with the paper.

Input data used in this study are from publicly available sources and referenced in Jennings et al. (2022). In summary, these consist of:
- The CDF-t bias-corrected CMIP5 data over Africa are available at http://amma2050.ipsl.upmc.fr/. To access the data, users must contact the lead author at moflod@locean-ipsl.upmc.fr.
- FAOSTAT yield and area and Food Balance Sheet data https://www.fao.org/faostat/
- Soil data were from the Regridded Harmonized World Soil Database v 1.2: https://www.fao.org/soils-portal/data-hub/soil-maps-and-databases/harmonized-world-soil-database-v12/en/
- Gridded area data from LUH2 (https://luh.umd.edu/) and WDPA (https://www.protectedplanet.net/en/thematic-areas/wdpa?tab=WDPA).

The iFEED website details result narratives and visualisations of crop model output: https://ifeed.leeds.ac.uk/

Research involving human participants, their data, or biological material

Policy information about studies with human participants or human data. See also policy information about sex, gender (identity/presentation), and sexual orientation and race, ethnicity and racism.

Reporting on sex and gender	No such data were collected.
Reporting on race, ethnicity, or other socially relevant groupings	No such data were collected.
Population characteristics	No such data were collected.
Recruitment	No such data were collected.
Ethics oversight	No such data were collected.

Note that full information on the approval of the study protocol must also be provided in the manuscript.

Field-specific reporting

Please select the one below that is the best fit for your research. If you are not sure, read the appropriate sections before making your selection.

☐ Life sciences ☐ Behavioural & social sciences ☒ Ecological, evolutionary & environmental sciences

For a reference copy of the document with all sections, see nature.com/documents/nr-reporting-summary-flat.pdf

Ecological, evolutionary & environmental sciences study design

All studies must disclose on these points even when the disclosure is negative.

Study description	This study uses an integrated assessment framework that combines process-based modelling of crops and land use allocation with assessment of nutrition security and trade in extreme years. We do not use statistical methods or collect primary data.
Research sample	No data collection for this study.
Sampling strategy	No data collection for this study.
Data collection	No data collection for this study.
Timing and spatial scale	No data collection for this study.
Data exclusions	No data collection for this study.

Reproducibility	No data collection for this study.
Randomization	No data collection for this study.
Blinding	No data collection for this study.

Did the study involve field work? ☐ Yes ☒ No

Reporting for specific materials, systems and methods

We require information from authors about some types of materials, experimental systems and methods used in many studies. Here, indicate whether each material, system or method listed is relevant to your study. If you are not sure if a list item applies to your research, read the appropriate section before selecting a response.

Materials & experimental systems

n/a	Involved in the study
☒	☐ Antibodies
☒	☐ Eukaryotic cell lines
☒	☐ Palaeontology and archaeology
☒	☐ Animals and other organisms
☒	☐ Clinical data
☒	☐ Dual use research of concern
☒	☐ Plants

Methods

n/a	Involved in the study
☒	☐ ChIP-seq
☒	☐ Flow cytometry
☒	☐ MRI-based neuroimaging

Plants

Seed stocks	n/a
Novel plant genotypes	n/a
Authentication	n/a
